# Supplementary material for: Synthetic magnetoelectric coupling in a nanocomposite multiferroic
Source: Sci Rep. 2015 Mar 13;5:9089. doi: 10.1038/srep09089 (PMC4357856; doi:10.1038/srep09089)
Supplement: Supplementary Information [file srep09089-s1.pdf]

Supporting Information

Synthetic magnetoelectric coupling in a nanocomposite multiferroic

P. Jain,[1] Q. Wang,[1] M. Roldan,[2] A. Glavic,[3] V. Lauter,[3] C. Urban,[4] Z. Bi,[1] T. Ahmed,[1] J. Zhu,[1] M. Varela,[2,3] Q. Jia,[1] M.R. Fitzsimmons*[1]

[1]Los Alamos National Laboratory, Los Alamos NM 87545; [2]Universidad Complutense de Madrid, Madrid Spain 28040; [3]Oak Ridge National Laboratory, Oak Ridge TN 37831; [4]University of California at San Diego, La Jolla CA 92093

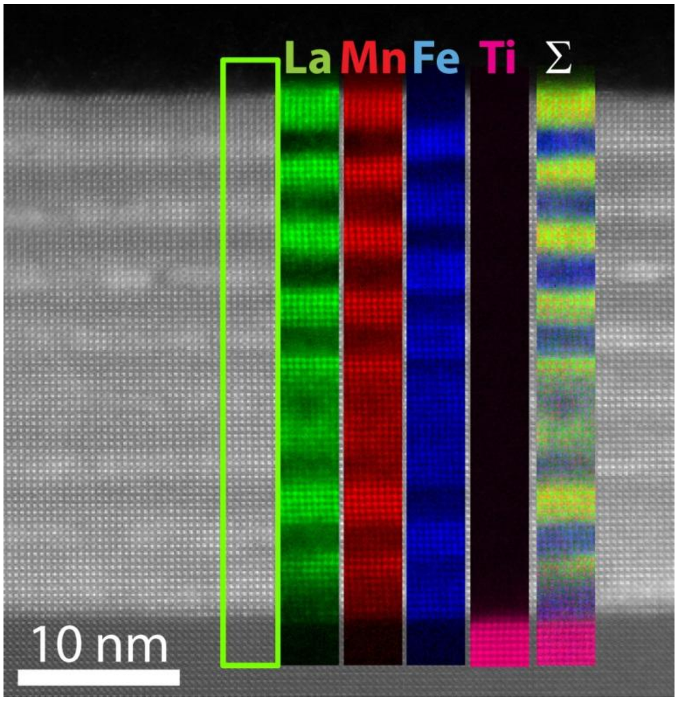

Fig. S1 Atomic resolution characterization in real space in real space was carried out by aberration-corrected scanning transmission electron microscopy (STEM). High angle annular dark-field (HAADF) Z-contrast imaging was combined with electron energy-loss spectroscopy (EELS). Observations were carried out in a Nion UltraSTEM200, equipped with a cold field-emission electron source and a fifth-order corrector, operated at 200 kV. EEL spectra were collected using a Gatan Enfinium spectrometer, the convergence semi-angle for the incident probe was 30 mrad, with an EELS collection semi-angle of 48 mrad. The figures shows a high-resolution HAADF image from the whole BFO/LSMO superstructure grown on a STO substrate. The green rectangle marks the area where an EEL spectrum image was acquired, with a dispersion of 0.25 eV per channel and a dwell time of 0.05 s per pixel. Together with the image there are EELS maps for the different elements. From left to right, La $M_{4,5}$ (green), Mn $L_{2,3}$ (red), Fe $L_{2,3}$ (blue) & *Ti* $L_{2,3}$ (magenta) maps together with the overlay.

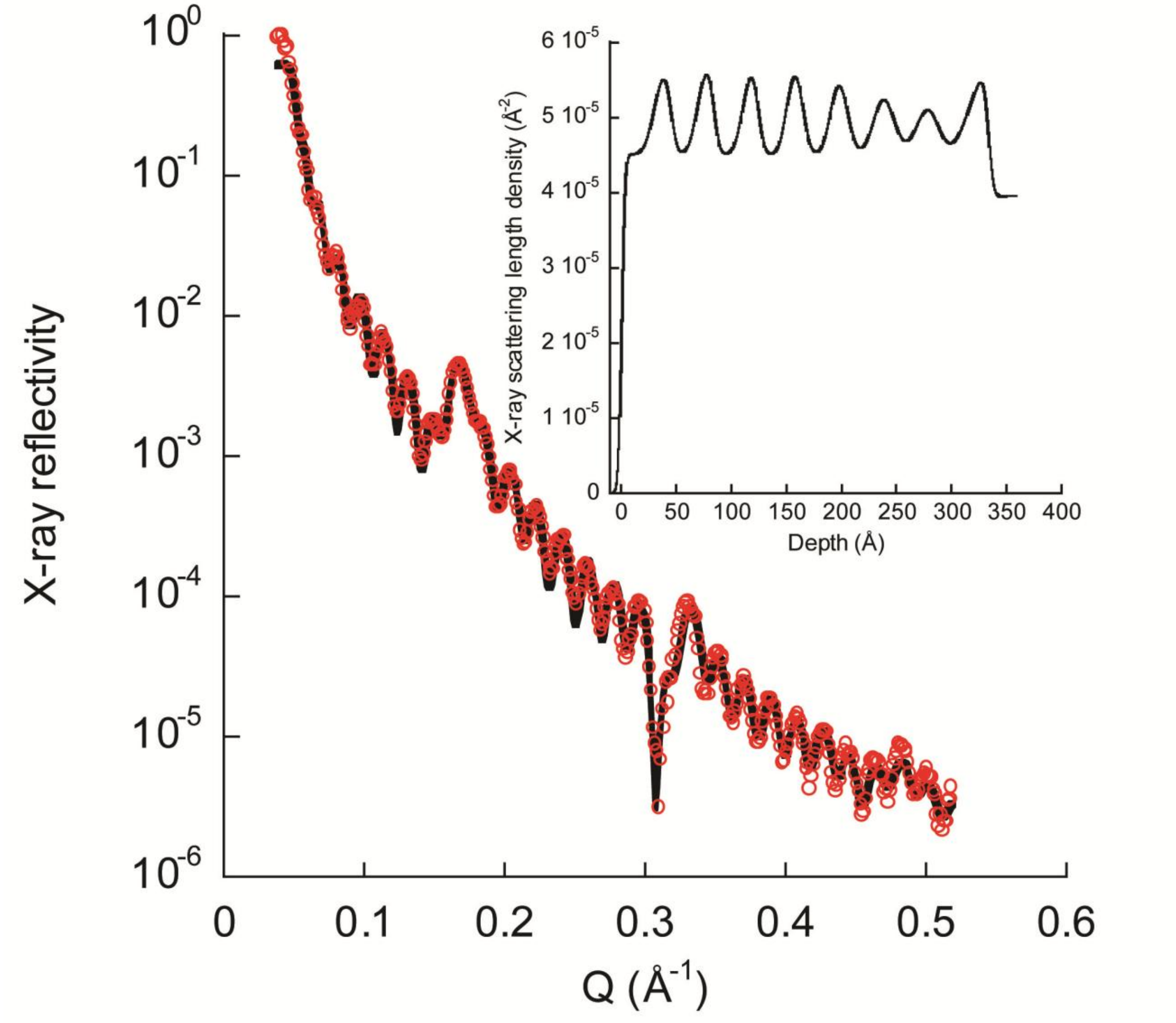

Figure S2 X-ray reflectivity of the superlattice sample (symbols). Inset: X-ray scattering length density profile of the superlattice from which the fitted reflectivity (black curve, main figure) was calculated.

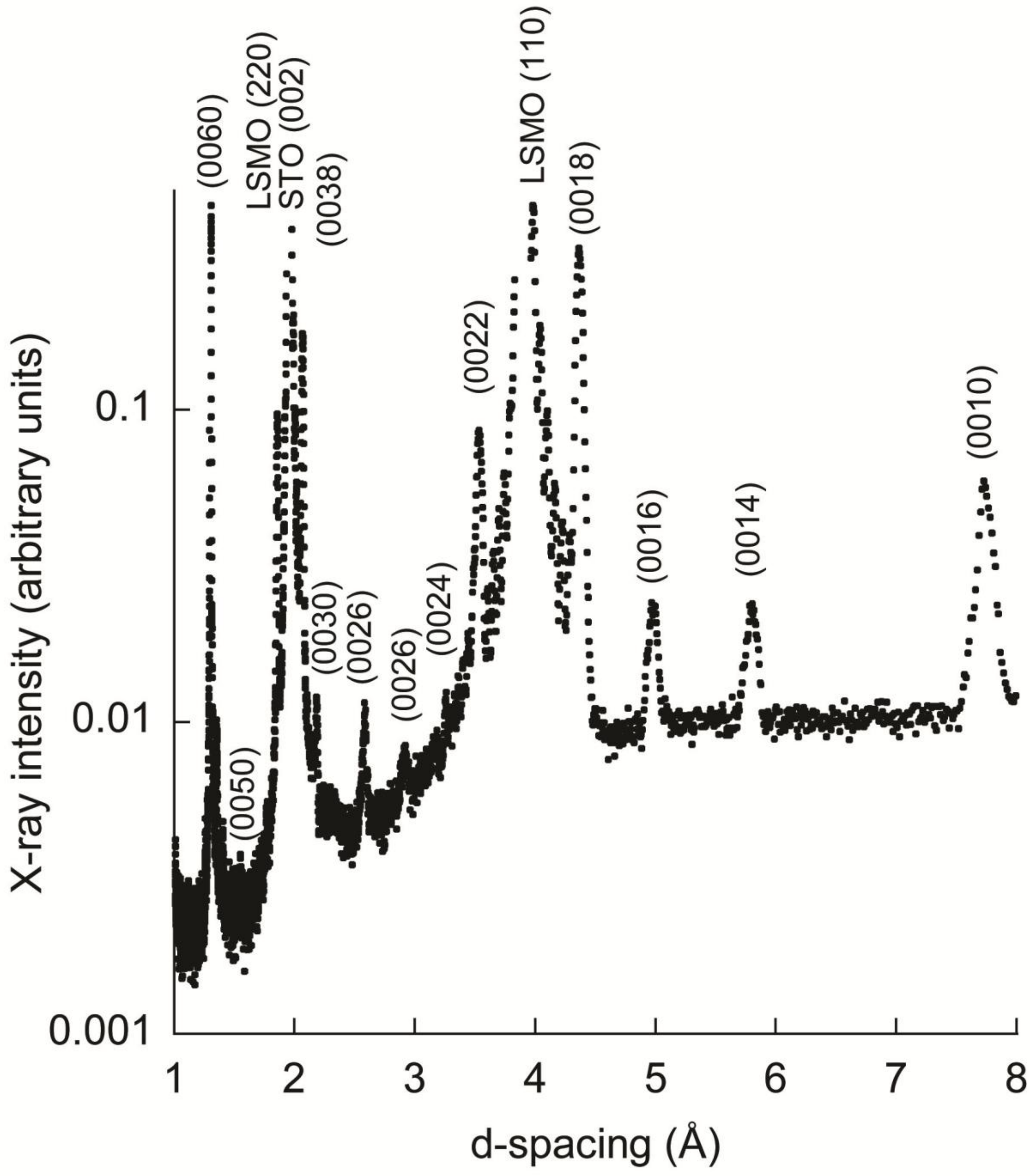

Figure S3 Out-of-plane x-ray diffraction pattern of the superlattice sample. Unlabeled index markers correspond to the order of the superlattice reflection. No distinction is observed between the lattice parameters of LSMO and BFO.

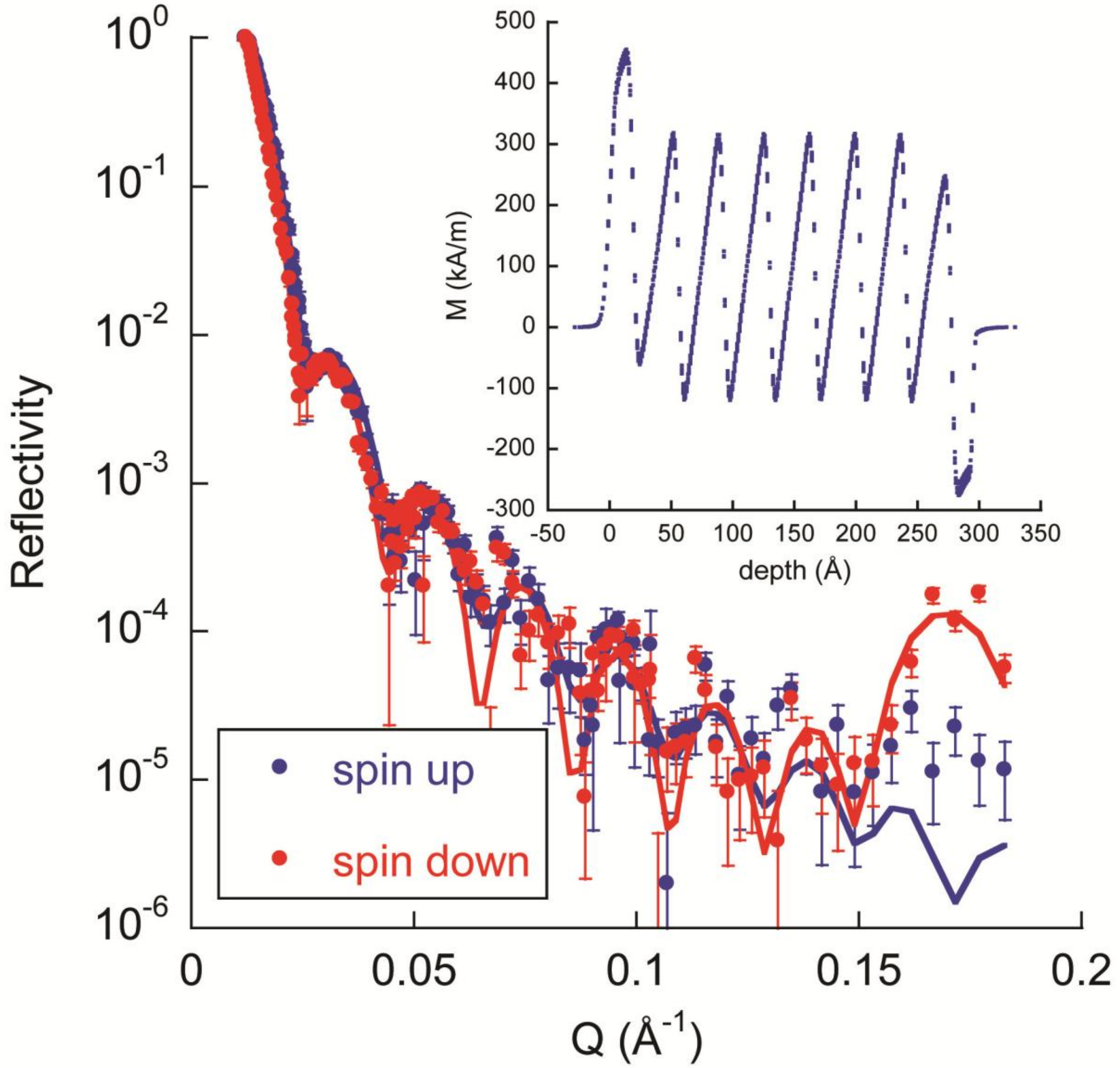

Figure S4 Neutron spin (up or down) reflectivities of the superlattice sample vs. wavevector transfer. The superlattice Bragg reflection is at Q ~ 0.18Å^{-1}. Inset: the magnetization depth profile obtained from the data. The reflectivity calculated from the magnetization and nuclear (not shown) profiles yield the solid curve in the main figure.

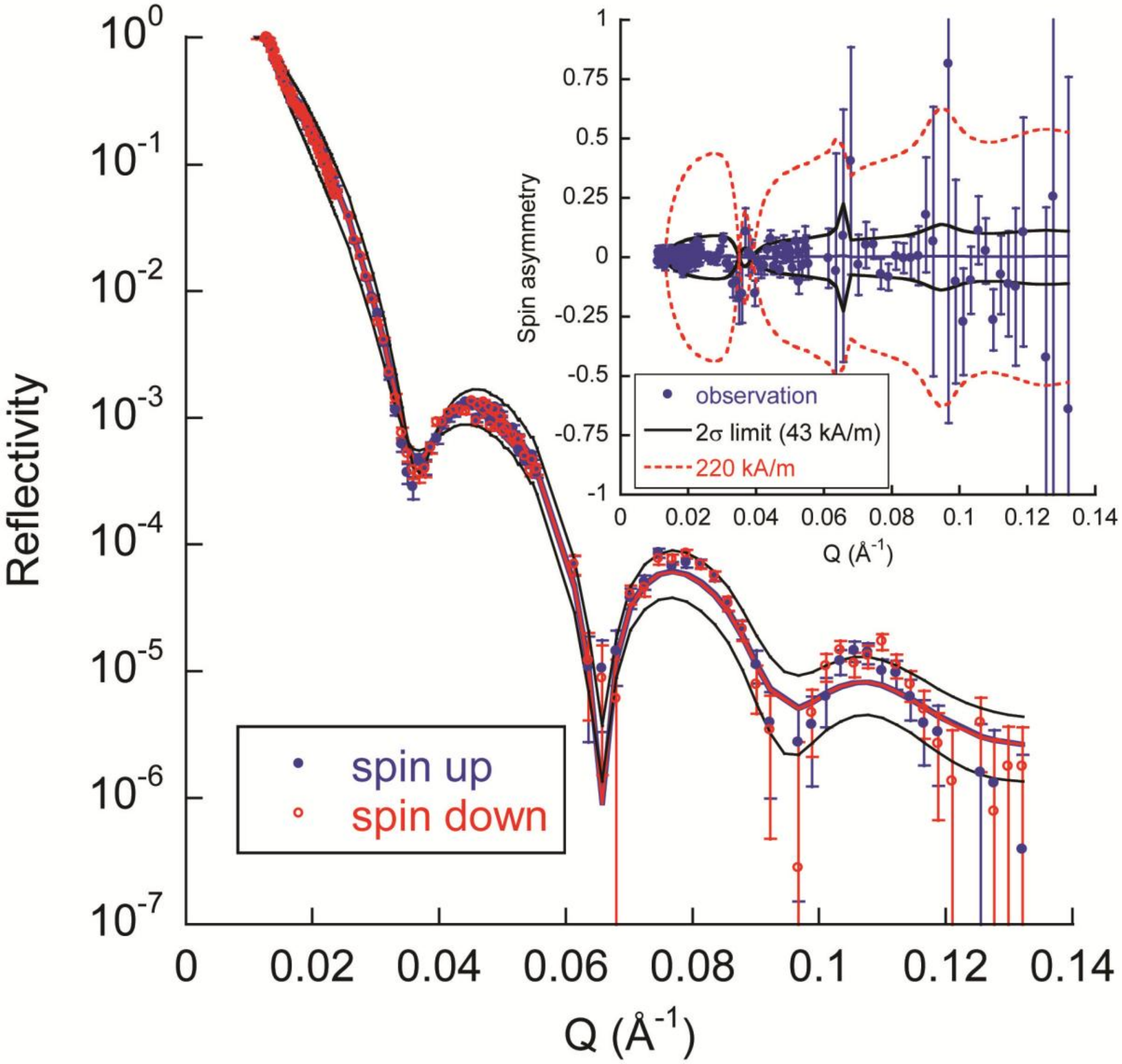

Figure S5 Neutron reflectivity of the 20 nm thick BFO film and (inset) spin asymmetry of the reflectivities (difference of the up and down reflectivities normalized by their sum). No evidence for uncompensated magnetization of the film was observed. The 2-σ limit on the maximum uncompensated magnetization of the film is 43 kA/m.

Methods

Polarized neutron reflectometry

Polarized neutron reflectometry (PNR) measurements of the superlattice and thick BFO film samples were performed at the Spallation Neutron Source and the Lujan Neutron Scattering Center, respectively. Briefly, PNR involves measurement of the specular reflectivity, R, of the sample as a function of wave vector transfer Q = $4\pi\sin\vartheta/\lambda$, where ϑ is angle of incidence between incident wavevector and its projection onto the sample's surface, and λ is neutron wavelength. The reflectivity is measured for polarized neutron beams with spin parallel (R^+) or opposite to (R^-) the applied field. The reflectivity is related to square of the Fourier transform of the spin-dependent scattering length density (SLD) depth profile $\rho^{\pm}(\mathrm{z}) = \rho_n(\mathrm{z}) \pm CM(\mathrm{z})$, where $\rho_n(\mathrm{z})$ is the nuclear scattering length density depth profile, C = 2.911×10^{-9} Å^{-2} m/kA, and M(z) is the magnetization (in kA/m) depth profile.

PNR measurements were made after first cooling the sample in a 5 kOe field to 10 K. The measurements were made as the sample was warmed to above ~170 K in the 5 kOe field. The measurements were fitted to a model in which the magnetizations of the BFO and LSMO layers were optimized to minimize the ^{2} metric for goodness of fit [P.R. Bevington D.K. Robinson, Data Reduction and Error Analysis in the Physical Sciences (3rd ed. McGraw-Hill, New York) (2003) p. 65]. $R^{\pm}$(Q) were calculated using the dynamical formulism of Parratt [Parratt, L. G. Surface studies of solids by total reflection of x-rays. *Phys. Rev*. **95**, 359-369 (1954)]. Error bars on the extracted magnetizations degrade the value of ^{2} by the number four (i.e., a 2-sigma error).

Capacitance measurement

Capacitance measurements were performed in a Quantum Design physical property measurement system using a home built probe at the national high magnetic field laboratory. AH 2500A, 1 kHz automatic capacitance bridge was used for accurate measurement of capacitance and loss. Magnetic field up to 12T was applied using the VSM.

Thin film growth

The composite targets used in this work were prepared by a conventional ceramic sintering process. PLD (KrF excimer laser, λ = 248 nm) was employed to grow nanocomposite films on STO (0 0 1) substrates. Substrate temperatures of 500–800 °C and suitable oxygen pressures of 10–350 mTorr were maintained during the film deposition. Following that, the samples were cooled down to room temperature in an oxygen-rich atmosphere (60–200 Torr) to ensure proper film stoichiometry.
